# Investigation of Dielectric Barrier Discharge Plasma for the Degradation of Erythromycin Solution

**DOI:** 10.3390/molecules30030625

**Published:** 2025-01-31

**Authors:** Yifan Liu, Xiaolong Wang, Zongzheng Wang, Tianao Xv, Xiaowen Dai, Yadi Liu, Ying Sun, Tong Zhao, Yuantao Zhang

**Affiliations:** School of Electrical Engineering, Shandong University, Jinan 250061, China; 202334728@mail.sdu.edu.cn (Y.L.); 202334777@mail.sdu.edu.cn (Z.W.); wq2103883976@163.com (T.X.); dxw@mail.sdu.edu.cn (X.D.); liuyadi@sdu.edu.cn (Y.L.); ys2018@sdu.edu.cn (Y.S.); zhaotong@sdu.edu.cn (T.Z.); ytzhang@sdu.edu.cn (Y.Z.)

**Keywords:** dielectric barrier discharge plasma, antibiotic degradation, erythromycin

## Abstract

Antibiotic contamination constitutes a serious environmental and public health risk. In order to fill the gap in the study of plasma degradation of erythromycin (ERY), this paper systematically investigated the mechanism of ERY degradation by dielectric barrier discharge (DBD) plasma. The underlying reaction mechanisms were investigated by experiments and molecular dynamics simulations. Plasma emission spectra revealed active hydroxyl radicals (·OH) and argon (Ar) spectral lines. The degradation efficiency of plasma treatment for ERY was found to be strongly influenced by treatment parameters, including applied voltage, treatment duration, and gas flow rate. In particular, a maximum degradation of 90% was achieved for a 250 mg/L ERY solution under conditions of 18 kV voltage, 850 sccm gas flow rate, and 60 min of treatment. The presence of ·OH and hydrogen peroxide (H_2_O_2_) in the reaction and their important role in the degradation were proved experimentally. Fracture of the ERY lactone ring induced by hydrogen abstraction reactions with reactive oxygen species (ROS) was observed by molecular dynamics simulations. In the in vitro antimicrobial assays targeting *Staphylococcus aureus*, the treated solutions demonstrated low toxicity, underscoring the practical applicability of dielectric barrier discharge (DBD) plasma technology in addressing antibiotic contamination in aquatic environments.

## 1. Introduction

The widespread use and application of antibiotics in both medical and veterinary fields have resulted in the release of over one million tons of these substances into the biosphere [1]. Notably, more than 80% of the administered antibiotics remain unabsorbed by animals, being excreted primarily via feces and urine. These excreted antibiotics end up in various environmental matrices, mainly in the aquatic environment, causing widespread environmental pollution [2,3]. This pollution is particularly prevalent in areas such as livestock farms and hospital wastewater effluents, where antibiotic residues pose a substantial risk of entering the food chain, thereby threatening the integrity of ecosystems [4]. Persistence and accumulation of these substances in the environment exacerbate the problem of antibiotic resistance in bacteria [5,6]. Research indicates that approximately 20% of antibiotics exhibit high toxicity to algae, while 44% demonstrate notable toxic effects on water fleas. Furthermore, over 50% of antibiotics present considerable biological toxicity risks to fish species [7]. The overuse of antibiotics, coupled with their detrimental environmental impact, has emerged as a critical challenge in contemporary global health and sustainable development [8]. A report published by the World Health Organization (WHO) on the monitoring of antibiotic use from 2016 to 2018 revealed that macrolides were the second most commonly used antibiotic [9]. In the research and investigation of antibiotics in groundwater in China, 28 commonly used antibiotics were detected at concentrations ranging from 0.1 to more than 1000 ng/L. The antibiotics with the highest frequency of detection were norfloxacin, ofloxacin, Sulfamethoxazole, sulfadiazine, enrofloxacin, and erythromycin (ERY) [10]. ERY has been flagged as a substance with the potential to harm aquatic ecosystems [11].

Several techniques have been proposed to mitigate antibiotic contamination, including biological treatment [12], advanced oxidation processes [13], and adsorption removal [14]. However, in practical application, the biological method requires high water quality, which can be easily damaged by improper use, the chemical oxidation method is prone to secondary pollution due to incomplete reaction, and the adsorption method is selective to pollutants, which makes it difficult to be applied in complex environments [12,13,14]. In recent years, plasma technology has attracted much attention and has emerged as a promising solution for the effective removal of pollutants from contaminated environments [15,16,17]. Plasma technology offers several advantages, such as short treatment times, high efficiency and environmentally friendly, prompting growing interest in its application for antibiotic removal [18,19,20,21,22]. The reactive oxygen species (ROS) produced during plasma treatment has been shown to have a degrading effect on a wide range of substances [23,24,25,26,27,28]. Studies by Fang et al. demonstrated the effectiveness of cold plasma jets at atmospheric pressure in removing norfloxacin and chloramphenicol from aqueous solutions, achieving removal efficiencies of 80.06% and 70.61%, respectively, within 16 min of treatment. The researchers also identified hydroxyl radicals (·OH) and singlet oxygen (^1^O_2_) as crucial contributors to the degradation process [23]. In another study, Fang et al. treated levofloxacin and sulfadiazine with dielectric barrier discharge (DBD) plasma and found that the highest removal efficiency was achieved when the oxygen-to-nitrogen ratio was optimized to 1:4. Additionally, the study indicated that ·OH and ozone (O_3_) played key roles in the degradation of these antibiotics [24]. Cheng et al. further enhanced the degradation efficiency of amoxicillin using a DBD plasma-catalyst synergistic system, demonstrating that O_3_ and hydrogen peroxide (H_2_O_2_) were essential in driving the reaction [28].

Plasma is generated in a variety of ways, mainly DBD [19], corona discharge [29], spark discharge [30] and glow discharge [19]. Corona discharges are produced by applying a high electric field around the sharp edges of a metal electrode using a DC, AC or RF power source, the energy of which is too concentrated at the tip of the electrode, resulting in a shorter electrode life and usually requiring a larger space to accommodate the electrode and corona layer formation area [29]. Spark discharge is the use of a high-voltage power supply to charge the capacitor bank, and then discharge through the capacitor between the two electrodes, the system produces a plasma with high energy density, but its ion source is unstable, prone to spark diffusion and arc instability and other problems, and the electrodes are susceptible to abrasion and wear and need frequent maintenance and replacement [30]. Glow discharge is a discharge phenomenon in which the working gas is penetrated at low pressure and shows a glow. The low electric field strength of the glow discharge process is also limited by the low air pressure and is therefore not utilised for industrial-scale applications. DBD is a discharge form that generates a high electron density and stable plasma in the discharge region by placing an insulating medium into the discharge space or covering the electrode surface. This configuration prevents localized arc discharge and electric sparks while ensuring the formation of a stable plasma. DBD plasma offers several advantages, including a wide range of applications, high electron density, long reactor lifespan, and effective wastewater treatment performance. As a result, it has garnered significant attention in the field of wastewater treatment [19]. In the application of DBD plasma discharges, researchers have found that the treatment is good for a wide range of antibiotics such as sulfamethoxazole [31], norfloxacin [32], sulfadiazine [33], tetracycline [34], and gentamycin [20].

Despite the promising results of plasma treatments for various antibiotics, research on the plasma degradation of ERY remains limited. To fill this gap, a series of experiments were conducted to investigate the interaction between DBD plasma and ERY. Instead of simply focusing on the macroscopic degradation rate of DBD plasma degradation of ERY, the present study systematically and comprehensively explores the processes related to plasma degradation of ERY, starting from the plasma diagnosis of the device. For the ERY degradation process, the study first explored the effects of treatment parameters (e.g., applied voltage, treatment time and gas flow rate) on ERY degradation. Solution diagnostics and active particle capture experiments were then performed to determine the role of active species in the degradation process. In addition, the mechanism of action of ROS on ERY molecules was analysed using molecular dynamics simulations and the dose effect of different ROS was explored. Finally, the toxicity of the treated solutions was assessed against ERY-sensitive *S. aureus* strains [35].

## 2. Results and Discussion

### 2.1. Characteristics of DBD Plasma

**Figure 1:**
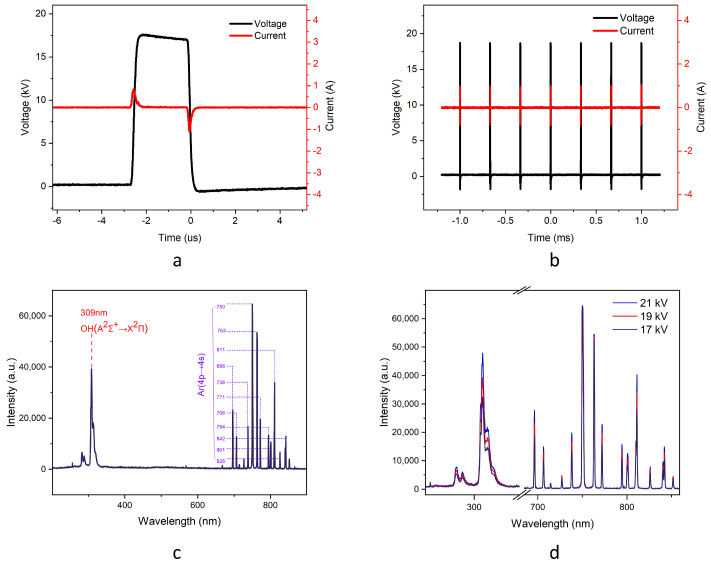
Plasma characteristics. (**a**) Multiple pulse waveforms; (**b**) Individual pulse waveforms; (**c**) 19kV emission spectrum; (**d**) Emission spectra at different voltages.

Figure 1a,b illustrate the waveforms of the discharge voltage and current of the experimental device when the peak output voltage of the pulse generator is 18 kV, the discharge frequency is 3 kHz and the pulse width is 2.4 µs. At the outset of the pulse, the air gap voltage rises continuously under the influence of the pulse generator source. The initial electrons situated in proximity to the cathode commence their movement towards the anode, the gas within the discharge gap undergoes gradual ionization, and the discharge current rises continuously until it reaches its peak. At this point, a considerable number of electrons accumulate on the surface of the dielectric barrier layer, resulting in the formation of a reversed electric field within the discharge gap. This, in turn, causes the air gap voltage to decline, leading to the gradual cessation of the ionization process. The discharge current then drops continuously until it reaches zero. As a consequence of the decline in pulse voltage, the reverse electric field generated by the accumulation of charge at the surface begins to strengthen, leading to a second discharge in the discharge gap. It can thus be demonstrated that the peak discharge current occurs at the rising and falling edges of the voltage. The rate of change of the pulse voltage exerts a significant influence on the discharge. A steeper rate of change of the pulse voltage results in a more intense DBD discharge [36,37]. By multiplying and integrating the observed current and voltage data, the total energy consumed during a single discharge pulse was found to be 0.577 mJ, with an average power of 1.733 W over the entire discharge cycle.

**Figure 2:**
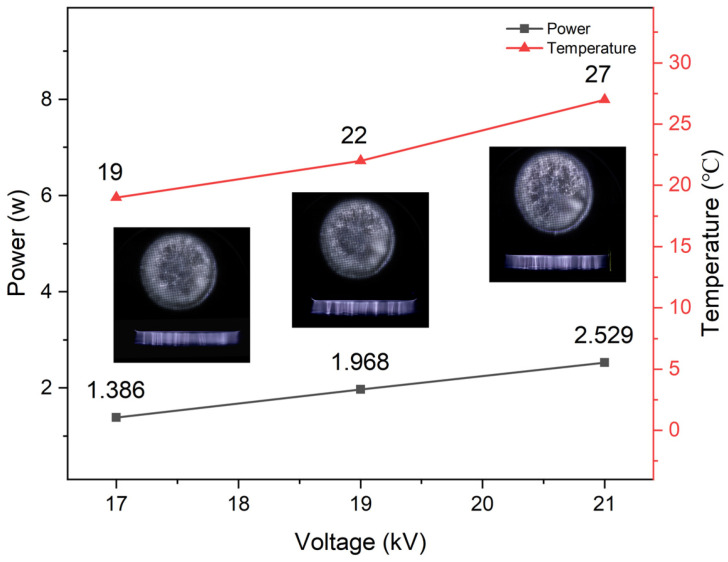
Solution temperature, discharge power and discharge image at different discharge voltages.

Figure 2 illustrates the variations in discharge images, discharge power, and the temperature of the reaction liquid (after a 60-min treatment) with respect to the applied voltage. The discharge images were captured from both the side and the bottom views. The discharge space is situated in the middle of the reaction liquid and the barrier medium. This configuration results in a higher volume fraction of H_2_O in the discharge region, which in turn causes an aberration of the spatial electric field. This results in reduced discharge uniformity, which subsequently causes the development of unstable discharge filaments within the device. Furthermore, the space luminescence exhibits a different luminance, which is prone to a cluster-like distribution [37]. As the voltage level rises, the number of discharge filaments increases, accompanied by an overall enhancement in the luminosity of the discharge space. An increase in the applied voltage from 17 kV to 21 kV resulted in a corresponding rise in discharge power, from 1.386 W to 2.529 W. Furthermore, at higher voltage levels, the accumulation of thermal energy was more significant, with the temperature reaching up to 27 °C.

Figure 1a,b illustrate the emission spectra of the discharge process, as observed from the lateral aspect of the device. Since the discharge region is located above the liquid phase, a unique emission spectrum of ·OH groups at a wavelength of 309 nm can be detected. Concurrently, discernible Ar atomic emission spectral lines were observed within the range of 696–842 nm [38,39,40]. With the increase in discharge intensity, the overall spectral intensity exhibits a corresponding rise. Notably, the intensity of the spectral lines near ·OH shows a marked enhancement. While the intensity of the argon Ar spectral lines also increases, the degree of enhancement is comparatively less significant when contrasted with the spectral lines near the hydroxyl group.

### 2.2. Analysis of Degradation Efficiency

**Figure 3:**
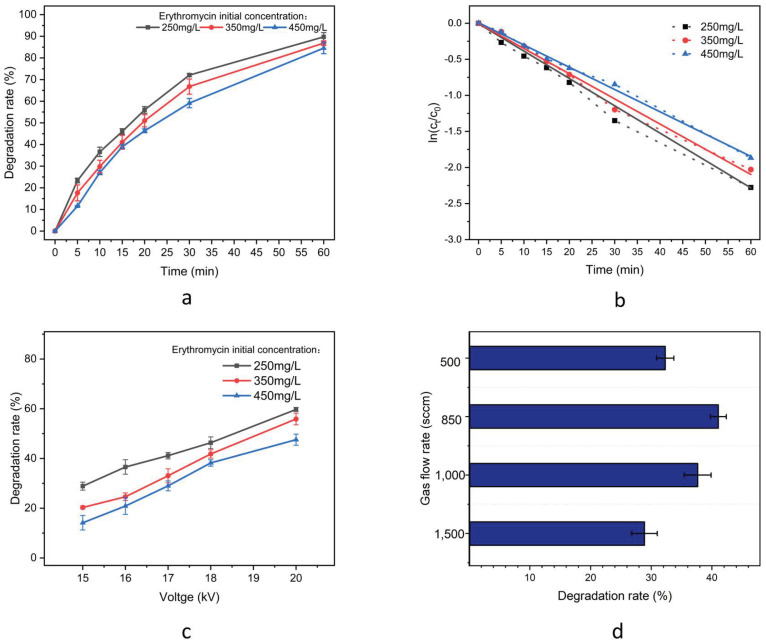
ERY degradation experiment. (**a**) ERY degradation rate as a function of concentration and time; (**b**) First-order kinetics of degradation (the solid line shows the fitted curve and the dashed line shows the actual data); (**c**) ERY degradation rate as a function of voltage; (**d**) ERY degradation rate versus gas flow rate.

To investigate the impact of plasma treatment time on the degradation of ERY, a series of experiments were conducted under identical discharge conditions (peak voltage of 18 kV, discharge frequency of 3 kHz, pulse width of 2.4 µs, and gas flow rate of 850 sccm). Three replications of each experiment were performed. In light of the analytical cost considerations and the detection sensitivity limits of the liquid chromatography system, the concentration gradient was optimized at 450 mg/L, 350 mg/L, and 250 mg/L. Additionally, the time gradient was established with the following intervals: 5 min, 10 min, 15 min, 20 min, 30 min, and 60 min. This design ensures sufficient resolution while accounting for the operational constraints of the equipment. At the end of the reaction, the pH was adjusted back to 7–8 with disodium hydrogen phosphate solution and the treatment solution was titrated to the initial volume. As illustrated in Figure 3a, the degradation rate (η) demonstrated an increase with the treatment time, irrespective of the initial concentration. As the reaction proceeds, the rate of reaction decreases. The experiment demonstrated that an ERY solution with an initial concentration of 250 mg/L reached a degradation rate of 90% after 60 min of treatment. This can be attributed to the fact that the quantity of ROS generated by the reaction apparatus increases with the duration of the treatment. As the reaction continues to operate, additional active material can be introduced into the solution, where it may collide with the ERY molecules, thereby facilitating a greater extent of ERY decomposition.

The initial sample concentration is a crucial factor in determining η. The η of the ERY solution with a higher initial concentration was found to be lower compared to that of the solution with a lower initial concentration, under identical treatment conditions. After five minutes of plasma treatment, η of the 250 mg/L ERY solution was 23.3%, while η of the 450 mg/L ERY solution was 11.5%. However, from the perspective of the number of molecules treated, a greater number of ERY molecules were degraded from the solution with the higher initial concentration. The ROS generated by the discharge reacted with ERY. Under the present discharge conditions, the ROS were more distributed near the discharge filaments. This resulted in a higher concentration of ERY molecules being in closer proximity to the ROS, particularly at the gas–liquid interface. Although high-concentration ERY solutions provide greater opportunities for localized interactions with ROS, from an overall perspective, achieving the same degradation rate at higher concentrations necessitates the degradation of a larger number of ERY molecules. Molecules added at high concentrations are not fully degraded, which leads to a decrease in the overall degradation rate. The degradation reaction kinetics of ERY solutions at varying initial concentrations were modeled, as illustrated in Table 1 and Figure 3b. This revealed that the observed concentration changes in the solutions were consistent with those predicted by a first-order reaction kinetics model.

Figure 3c shows the η of ERY as a function of applied voltage for different initial concentrations. The power supply frequency was 3 kHz, the pulse width was 2.4 µs, the gas flow rate was 850 sccm, and the treatment time was 15 min. Increasing the applied voltage significantly enhances the degradation effect on ERY. A comparable phenomenon was observed in the plasma treatment of other pollutants [41]. The η of a 450 mg/L ERY solution was 14.2% under 15 kV voltage treatment, while it reached 47.7% under 20 kV voltage treatment. The magnitude of the applied voltage exerts a significant influence on the discharge intensity within the reactor. As illustrated in Figure 2, at low voltage, the discharge filaments span a limited portion of the solution, and an increase in voltage leads to the generation of more discharge filaments within the discharge gap, thereby enhancing the η.

Furthermore, the rate of gas flow was observed to exert an influence on the extent of degradation. The impact of gas flow rate on degradation efficiency is multifaceted. On the one hand, an increase in gas flow rate results in a greater excitation of Ar into energetic particles within a certain range. However, when the gas flow rate reaches a certain critical value, the high concentration of Ar becomes more prone to the recombination of active Ar species, resulting in a decreased overall degradation efficiency. On the other hand, the gas flow rate affects the mobility of ROS from the gas–liquid interface to the interior of the liquid. When gas flow increases, the propagation direction of ROS changes from linear flow along the depth direction to vortex flow, which will result in a decrease in the degradation efficiency of the impact plasma [42]. Figure 3d illustrates the η of 250 mg/L ERY at a power supply frequency of 3 kHz, a pulse width of 2.4 µs, an electric potential of 18 kV, and varying gas flow rates over 15 min. The experimental data reveal that the η initially increases within a specific range before subsequently decreasing. Furthermore, the η is observed to be higher when the gas flow rate is approximately 850 sccm.

### 2.3. Solution Particle Measurement and Capture

**Figure 4:**
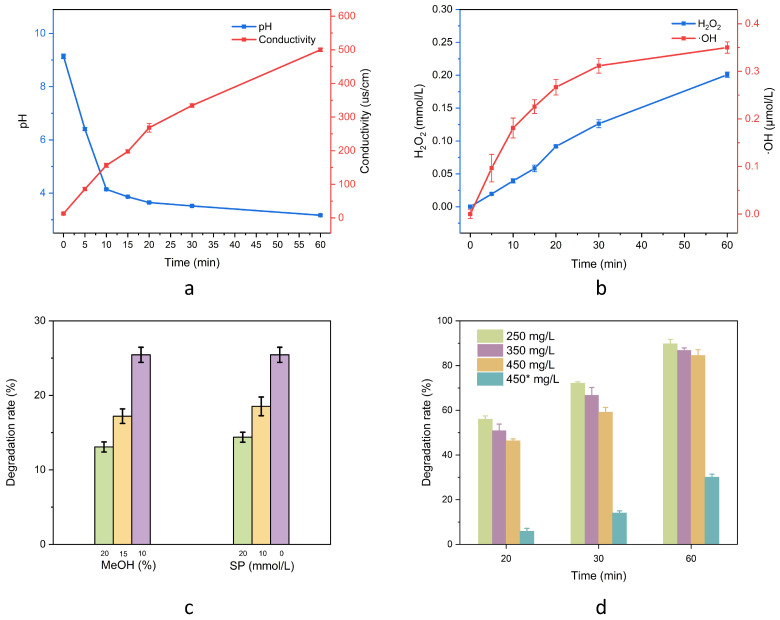
Solution detection and control experiments. (**a**) Changes in pH and conductivity σ at different treatment times; (**b**) Concentration of ·OH and H_2_O_2_ at different treatment times; (**c**) Effect of different concentrations of MeOH and SP on ERY degradation; (**d**) Acid degradation control (450* mg/L indicates acid degradation control).

In order to investigate the changes of active particles in the solution during the plasma treatment of ERY, the changes in pH and conductivity (σ) with plasma treatment time were measured in a solution of 450 mg/L ERY with a discharge voltage of 18 kV, a power supply frequency of 3 kHz, a pulse width of 2.4 µs, and a gas flow rate of 850 sccm. The solution shown in Figure 4a is weakly basic at first. After the plasma treatment, the initial pH value decreased rapidly to 6.4 within a five-minute interval. Thereafter, the trend was nonlinear, and the rate of change slowed down with the extension of the treatment time. Ultimately, the pH value decreased to 3.07 after 60 min of treatment, exhibiting a tendency towards stabilization. The observed decline in pH is primarily attributable to the increase in hydrogen ion concentration. Furthermore, it facilitates the decomposition of ERY in an acidic environment. As the treatment time was extended, the σ of the solution increased from 12.89 µs/cm at the outset to 501.43 µs/cm at the conclusion. This occurrence can be explained by two main factors: firstly, the active particles present in the solution increased in number as the reaction proceeded; secondly, the small molecules produced during the decomposition of ERY also contributed to the overall σ of the solution.

To exclude the effect of pH and temperature, the 450 mg/L ERY solution was titrated to the corresponding pH by phosphoric acid before each reaction node. The results are shown in Figure 4d. It was found that the ERY solution started to have an obvious degradation effect after 20 min, i.e., the pH was less than 4, and the acid degradation effect became more obvious with the decrease in pH.

In plasma treatment, energetic electrons and ROS generated by the discharge gas are transferred to the liquid phase through the gas–liquid interface, where a series of reactions occur, resulting in alterations to the solution state and active particles. It is anticipated that both long-lived and short-lived oxidizing substances, in addition to the energetic electrons generated in the discharge, will participate in the decomposition process and contribute to the overall outcome. The alterations in the content of H_2_O_2_ and ·OH in deionized water were examined under the conditions of a discharge voltage of 18 kV, a power frequency of 3 kHz, a pulse width of 2.4 µs, and a gas flow rate of 850 sccm. As shown in Figure 4b, the experiment showed that the content of H_2_O_2_ and ·OH in solution increased significantly. After 30 min of treatment, the concentration of H_2_O_2_ reached 0.20 mmol/L, while the concentration of ·OH reached 0.35 µmol/L. The relevant reaction mechanisms are shown in Table 2 [43,44,45].

To further investigate the effect of ·OH and H_2_O_2_ on the degradation efficiency during the reaction, methanol (MeOH)and sodium pyruvate (SP) were added to the experimental solution as ·OH and H_2_O_2_ trapping reagents. This was carried out to investigate the involvement of ROS in the degradation of ERY, as shown in Figure 4c. These reagents were observed to react with ROS in solution, thereby competing with antibiotic molecules. The experiments demonstrated a notable reduction in the degradation of ERY following the treatment of 450 mg/L ERY solution for 10 min with a discharge voltage of 18 kV, a power frequency of 3 kHz, a pulse width of 2.4 µs, a gas flow rate of 850 sccm, and the capture agent of MeOH and SP. As shown in Figure 4c, the addition of 20 mmol/L SP resulted in an 11.06% reduction in the degradation efficiency of ERY. Furthermore, the η in a 20% MeOH aqueous solution was observed to decrease by 12.37% in comparison to a 10% MeOH aqueous solution. This suggests that both ·OH and hydrogen peroxide played a significant role in the experimental process.

### 2.4. Possible Reaction Pathways Between ROS and ERY

**Figure 5:**
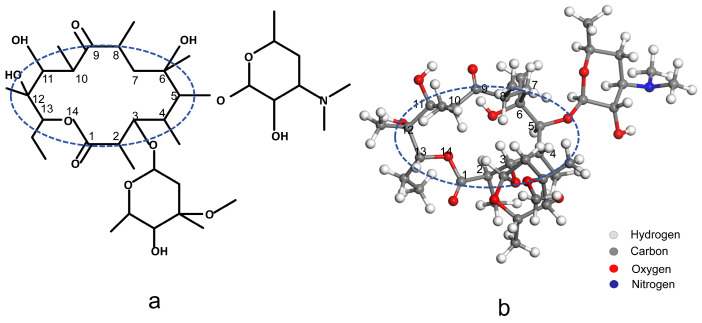
Structure of ERY (**a**) chemical structure and (**b**) simulation model.

The macrolide core, a defining structural feature of macrolide antibiotics, typically comprises a macrolactone ring containing 12 to 16 carbon atoms. This core plays a critical role in the antibiotic’s mechanism of action, facilitating its binding to bacterial ribosomes. Such binding is essential for the antibiotic’s efficacy, as it effectively inhibits bacterial protein synthesis [46]. In Figure 5, this macrolide core is highlighted within a blue circle to underscore its structural and functional significance. In practical applications, macrolides frequently lose their antimicrobial efficacy due to the degradation of their core structure. For instance, the bacterial strain Ochrobactrum WX-J1 has been shown to degrade ERY A through sequential processes such as deglycosylation and cleavage of the lactone bond, ultimately breaking it down into simpler compounds like propionic acid and carbon dioxide [47]. Similarly, ERY esterases catalyze the hydrolysis of the macrolide ring, thereby rendering the compound inactive as an antimicrobial agent [48,49]. Selection of ·OH and H_2_O_2_ observed in previous studies for molecular dynamics simulations. During the reaction, the macrolide core of ERY was effectively degraded, providing clear evidence of the capability of ROS to induce structural damage to ERY and facilitate its breakdown.

**Figure 6:**
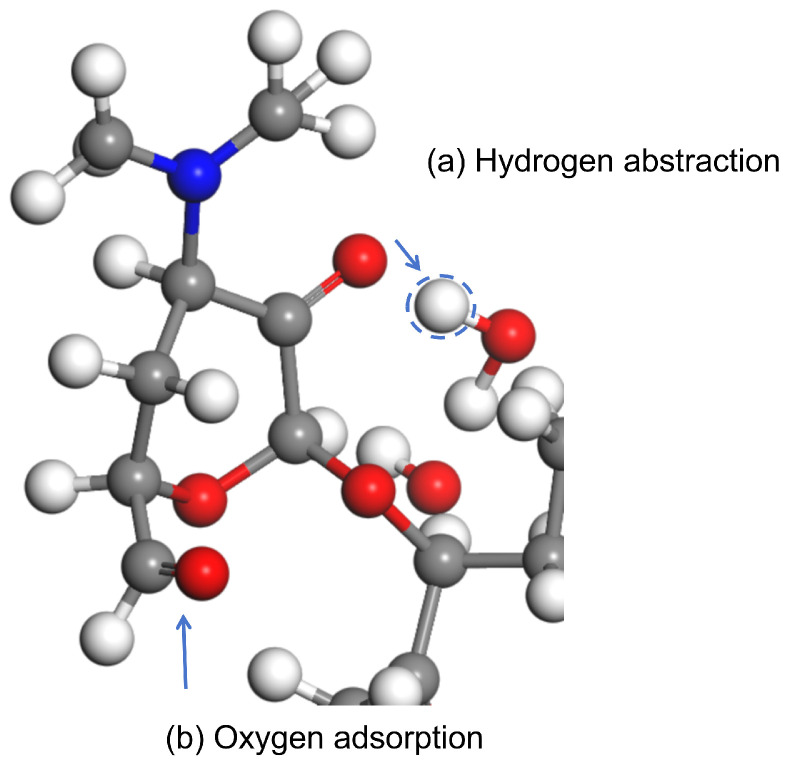
Examples of reactions. (**a**) Hydrogen abstraction and (**b**) Oxygen absorption.

**Figure 7:**
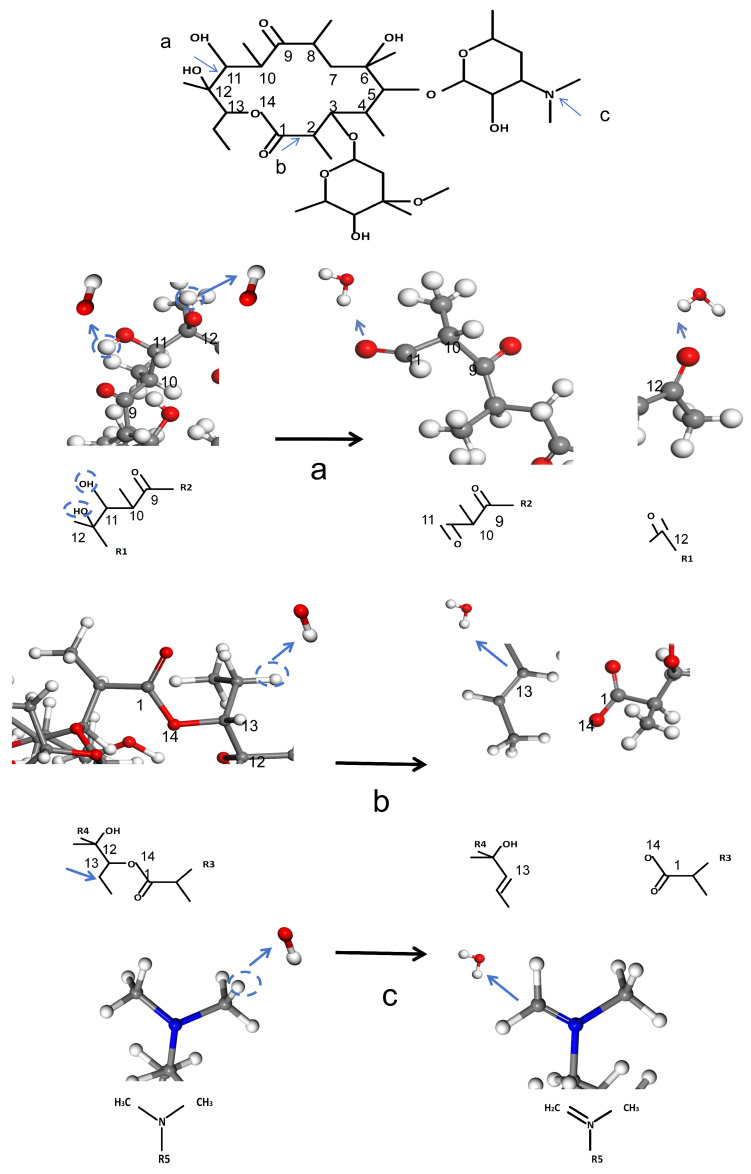
ROS attacks ERY process.

The simulation operates within a canonical ensemble (NVT), maintaining a constant number of atoms, fixed volume, and stable temperature. The simulation environment is idealized as a perfect vacuum. During the process, the majority of ROS participate in hydrogen abstraction or adsorption reactions, as illustrated in Figure 6. These reactions subsequently result in the cleavage of C-O and C-C bonds, alongside the formation of new C=O and C=C bonds. The three primary reactions are illustrated in Figure 7. R1–R5 are the ignored parts in the figure. Reaction (a) involves the abstraction of hydrogen atoms from the hydroxyl groups at the C11 and C12 positions, resulting in the formation of a C=O double bond. This transformation subsequently induces the cleavage of the C11-C12 single bond. The formation of C=O double bonds at the C11 site was reported in Product 8 in the study of Pishrafti et al. [50]. Reaction (b) involves the abstraction of the branched hydrogen atom from the C13 position, resulting in the formation of a C=C double bond between C13 and the branched chain. This process is accompanied by the cleavage of the single bond between C13 and O14. The final formation of the carboxyl group at the C1 site was reported for compound D in the study of Luiz et al. [51] and for Product 4 in the study of Pishrafti et al. [50]. There is also reaction (c), where ERY is susceptible to ROS attack due to its tertiary amine group with a lone valence electron, which can directly attack the dimethylamino group, leading to the formation of C=N, and the possibility of further hydrolysis of this functional group to form formaldehyde and methyl exfoliation, which has also been mentioned in the studies of other people [51]. Product 8, Compound D, and Product 4 are represented in Table 3.

**Figure 8:**
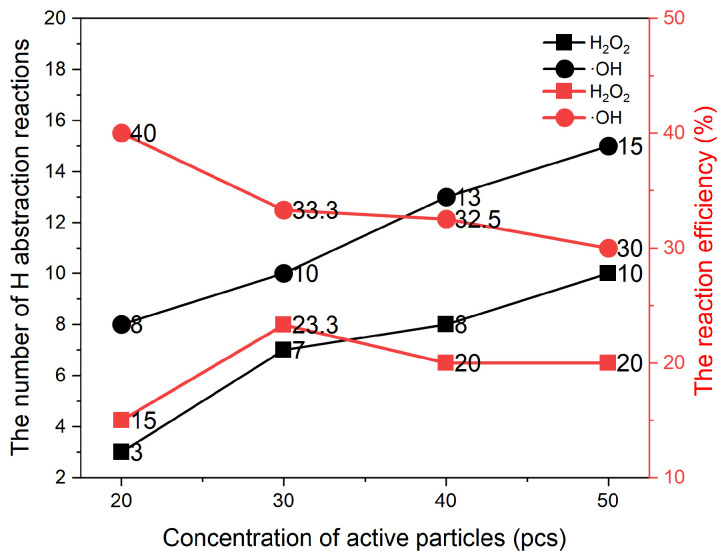
The relationship between the average number of occurrences of hydrogen abstraction, the efficiency of occurrence and the concentration of active particles.

The hydrogen abstraction reaction is the main reaction that triggers structural damage. Figure 8 analyses the relationship between the average number of occurrences of hydrogen abstraction, the efficiency of occurrence and the concentration of active particles. The average number is the average of the data of three simulated occurrences of hydrogen abstraction reactions for each ROS at the same concentration level, and the efficiency is the ratio of the average number of hydrogen abstraction reactions to the ROS in the cell. The number of hydrogen abstraction reactions increases as the concentration of active particles increases, and ·OH and H_2_O_2_ increase at the same rate, and it can be clearly seen that the reactivity of ·OH is greater than that of H_2_O_2_. The hydrogen abstraction efficiency of ·OH decreases with the increase in concentration, and the hydrogen abstraction efficiency of H_2_O_2_ shows a tendency to increase and then decrease, and the hydrogen abstraction efficiency of ·OH is higher than that of H_2_O_2_. The decrease in hydrogen abstraction efficiency can be interpreted as the limited number of sites in the ERY that can react, and the sites that are easy to react have already reacted at low concentrations, and this increased portion of ROS will not all participate in the reaction.

### 2.5. Results of In Vitro Antimicrobial Activity Assay

**Figure 9:**
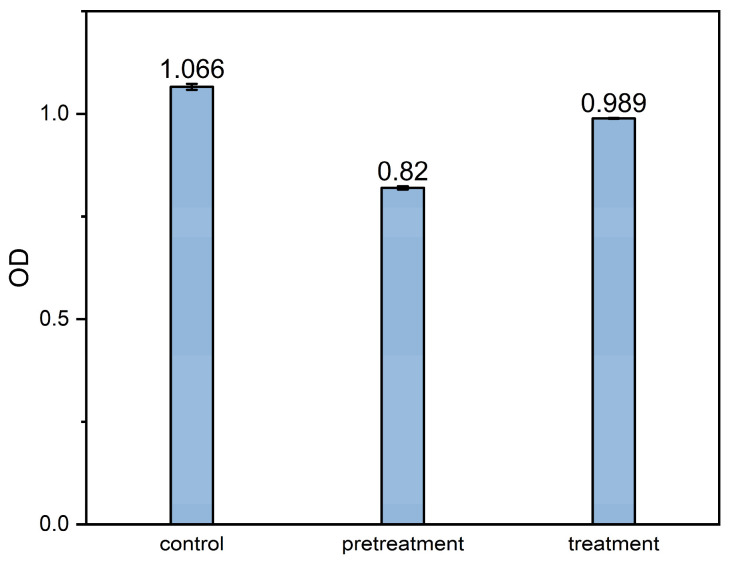
Results of antibacterial activity in vitro.

The safety of plasma-degraded ERY products was evaluated through in vitro antimicrobial assays against *Staphylococcus aureus*. As illustrated in Figure 9, the control group without experimental samples exhibited an average optical density (OD) value of 1.066. Upon direct addition of the untreated ERY solution, the average OD value decreased to 0.82, reflecting a 23% reduction and demonstrating significant inhibition of *S. aureus* growth. In contrast, the plasma-treated ERY solution (treatment parameters: initial ERY concentration of 450 mg/L, 18 kV, 3 kHz, 2.4 μs, 850 sccm, 30 min) resulted in an average OD value of 0.989, indicating a substantial reduction in inhibitory efficacy compared to the untreated ERY solution. These findings suggest that plasma treatment does not generate additional harmful by-products that adversely influence bacterial growth, underscoring the potential practical applicability of plasma treatment for ERY modification.

## 3. Materials and Methods

### 3.1. Chemicals

All chemicals used herein are of analytical grade or better. ERY, methanol (MeOH), ethanol (ET), phosphoric acid (H_3_PO_4_), acetonitrile (ACN), dipotassium hydrogen phosphate (DKP), titanium sulfate (TiOSO_4_), methylene blue (MB) and sodium pyruvate (SP) were produced by Macklin, Shanghai, China.

### 3.2. Experimental Setup

**Figure 10:**
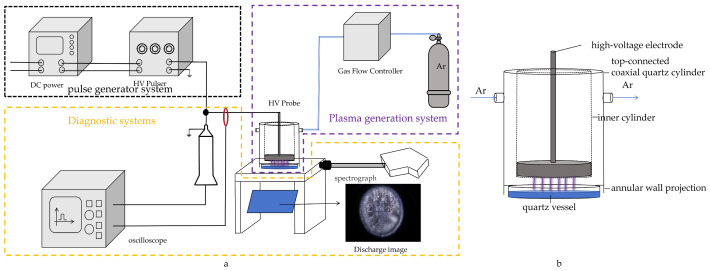
Experimental platforms. (**a**) Schematic diagram of the experimental setup; (**b**) DBD reactor.

Figure 10 illustrates a schematic diagram of the experimental setup, featuring the pulse generator system, plasma generation system, and diagnostic system. The pulse generator system comprises a low-voltage direct current (DC) power supply (Hansheng Puyuan Technology Co., Ltd., Beijing, China, HSPY1000) and a Marx generator (developed by Professor Lu’s team at the School of Electrical Engineering, Huazhong University of Science and Technology). The generator can generate a pulsed high voltage with a maximum amplitude of 30 kV, a pulse width of 1.3–20 µs, and a repetition frequency of 1–4.5 kHz. The power supply uses the principle of capacitor charging in parallel and discharging in series to lift a low DC voltage to a pulsed high voltage, which can be verified by design using literature methods [52,53]. The plasma generator system is comprised of two main components: a plasma reactor and a gas control system. The upper part of the plasma reactor is a top-connected coaxial quartz cylinder with an outer cylinder diameter of 70 mm and a length of 108 mm with air inlet and outlet ports, the inner cylinder with a diameter of 50 mm and a length of 100 mm, and an annular wall projection at the bottom of 8 mm from the bottom, and the lower part of the plasma reactor is a quartz vessel with an outer height of 10 mm and an outer diameter of 60 mm. The high-voltage electrode was inserted from above in the experiment, and the entire reactor was placed in sequence on a fine wire mesh and indium tin oxide (ITO) film, with the wire mesh grounded. The working gas was argon (99.999%), and a gas flow control (Sevenstar Electronics Co., Ltd., Huzhou, China CS200A) meter regulated the gas flow rate. The reaction solution was placed in the quartz container. The application of the high voltage to the high-voltage electrode results in the excitation and ionization of the Ar within the reactor, facilitated by the dielectric barrier effect of the quartz glass situated beneath the electrode. This process creates plasma, resulting in the production of a significant amount of ROS [54]. The diagnostic system consisted of electrical and optical diagnostic systems. Discharge voltage and current waveforms were captured using a high-voltage probe (Tektronix, Beaverton, OR, USA P6015A, 1:1000) and a current probe (Magnelab, Boulder, CO, USA, CT-C2.5-SMA, calibration scale: 2.5V/A), and data were recorded using a digital oscilloscope (Rohde and Schwarz; Munich, Germany, RTM3004). The plasma was analyzed by emission spectroscopy. Optical signals were captured using a UV-Vis lens (diameter: 10 mm; focal length: 10 mm) and fiber optic connectors. A portable UV-visible emission spectrometer (BW Tek, Beijing, China, Exemplar LS, 200–900 nm) was used to quantify the emission spectrum versus wavelength. A reflector with a tilt angle of 45 degrees was mounted in proximity to the reactor in order to facilitate observation of the plasma within the reactor. Shot from the side and bottom with a camera (Nikon Corporation, Tokyo, Japan, D7100). Shooting parameters: aperture value f/3.8, exposure time 1/3s, ISO-100, exposure compensation +0.3, maximum aperture 3.9.

### 3.3. Analytical Methods

The concentration of the ERY solution was analyzed by high-performance liquid chromatography (HPLC) (Dalian Elite Analytical Instruments Co., Ltd., Dalian, China, EClassical D3200) using a 5 µm column (Dalian Elite Analytical Instruments Co., Ltd., Supersil ODS2). The mobile phases comprised acetonitrile (A) and dipotassium hydrogen phosphate (B, 0.01 mol/L), with a volume ratio of VA:VB = 55:45. The injection volume was 10 µL, the flow rate was 0.6 mL/min, the column temperature was 35 °C, and the detector wavelength was 210 nm, which was detected by an ultraviolet detector [55]. To observe the effect of plasma degradation in a significant manner, ERY was dissolved in 10% MeOH in water for treatment in this experiment. To characterize the degradation of ERY by plasma, we defined the degradation rate (η) of ERY which was calculated as follows:

(6) $$\eta =\frac{C_{t}-C_{0}}{C_{0}}\times 100\%,$$

where $C_{0}$ denotes the initial concentration of ERY solution (mg/L), and $C_{t}$ denotes the concentration of ERY solution (mg/L) after treatment t min.

As evidenced by studies on the degradation of other pollutants, regression analysis of the degradation change curve using the exponential rate model can yield favorable results [41,56,57]. The relationship expression is as follows:

(7) $$V=\frac{dC}{dt}=kc^{n},$$

where k is called the degradation rate constant, c is called the substrate concentration, and n is the number of reaction levels. The general reaction level number n = 1 is very common, and the treatment conditions in this experiment are similar to most of the first-level reaction conditions, and the plasma degradation of ERY in this experiment should be consistent with the 1st order reaction [41]. The experimental data were analyzed using a pseudo-primary kinetic model, and the rate constant was determined by the equation:

(8) $$ln(\frac{C_{t}}{C_{0}})=kt,$$

### 3.4. Active Substance Testing and Capture Experiments

Acidity meter (Shanghai Youke Instrumentation Co., Ltd., Shanghai, China, P109) and conductivity meter (Shanghai Yidian Scientific Instrument Co., Ltd., Shanghai, China, DDS-307A) were chosen to measure the conductivity σ and pH of the treated solution, and the data were recorded after waiting for the data to stabilise and repeated three times. The acid control experiments were performed as follows: the vessel was placed in a water bath at 30 °C and the solution was titrated with 10% phosphoric acid to a pH of 6.4 (corresponding to the pH after 5 min of plasma treatment) at 0 min, and then continued to titrate to a pH of 4.5 (corresponding to the pH after 10 min of plasma treatment) at 5 min, and so on. At the end of the reaction, the pH was adjusted back to 7–8 with disodium hydrogen phosphate solution and the treatment solution was titrated to the initial volume.

The content of hydrogen peroxide (H_2_O_2_) was determined through the reaction of TiOSO_4_ with H_2_O_2_. In the presence of acidic conditions, TiOSO_4_ reacts with H_2_O_2_, forming yellow titanium peroxide (IV) complexes. The complex was observed to remain stable in an aqueous solution, with shade of color demonstrating a functional relationship to the content of H_2_O_2_ present in the liquid phase. The maximum absorption peak was recorded at 407 nm [58]. ·OH content was quantified using an aqueous methylene blue solution. The MB cation (dark blue) underwent a reaction with ·OH, forming hydroxide ions and MB radical cations (colorless). This resulted in a linear correlation between the transparency of the MB solution and the concentration of ·OH. The maximum absorption peak for the MB solution was observed at 665 nm. The concentration of ·OH can be calculated by adding a methylene blue solution to the treated solution and measuring the change in absorbance before and after plasma treatment [59]. Moreover, the methylene blue staining assay demonstrated that the reaction of methylene blue cations with hydroxyl radicals remained unaffected by the presence of liquid-phase H_2_O_2_ in accordance with the method [60]. The monohydric alcohol has been demonstrated to exert a significant quenching effect on the ·OH. The impact of the ·OH on the degradation process was investigated by varying the MeOH content in the reaction system [61]. The impact of H_2_O_2_ on the degradation process was investigated by introducing SP into the reaction system to facilitate the capture of hydrogen peroxide [38,62].

### 3.5. Molecular Dynamics Simulation Platform

To investigate the reaction mechanisms underlying the interactions between plasma and EYR, the Materials Studio platform was employed to simulate the reaction dynamics between ROS and ERY using the ReaxFF reactive force field framework. The aforementioned simulation platform has previously demonstrated considerable accuracy in the research conducted by the group, as evidenced by references [41,54,63,64,65]. In the simulation, the ERY drug model is initially constructed, and the information regarding bond length, bond angle, and dihedral angle is adjusted through the clean module. Subsequently, the structure that most closely approximates the actual situation is identified through the processes of geometrical optimization and kinetic optimization. Finally, the common ROS (·OH, H_2_O_2_) are investigated by constituting the reactive cell together with the ERY molecule.

### 3.6. In Vitro Antimicrobial Activity Assay Method

Determination of in vitro antimicrobial activity was performed to determine the effect of plasma treatment and to verify the toxicity of the products. The ERY solution before and after the treatment was diluted to the corresponding working concentration (0.4 mg/L) using diluted *Staphylococcus aureus* bacterial solution, and incubated in an incubator at 37 °C for 18 h. A control group was set up without the addition of ERY solution. Then, 200 μL of the co-culture solution was introduced into the 96-well plate, and the optical density (OD) of each group was measured at 600 nm using an enzyme-linked immunosorbent assay reader.

## 4. Conclusions

In this paper, the DBD plasma degradation process of ERY is systematically studied by combining experiments and simulations, which fills the current gap in the research field of ERY plasma degradation, and provides technical methods and theoretical support for practical applications. In this paper, the DBD plasma reaction device was first constructed and the plasma itself was characterised. Optical photography showed many discharge filaments in the discharge space, and their emission spectra showed distinct -OH and Ar spectral lines. This system was effectively utilized to treat the degradation of ERY solutions. The results showed that the degradation rate of 250 mg/L ERY could reach 90% at 60 min. The effects of treatment time, applied voltage, and gas flow rate on ERY degradation were investigated. The degradation rate of the ERY solution increases with increasing plasma treatment time and applied voltage. The initial concentration of ERY determines the potential amount of ERY to be degraded by plasma. ERY molecules in highly concentrated solutions are more likely to come into contact with ROS substances, but the overall degradation rate becomes lower. The gas flow rate causes the ERY degradation to increase first and then decrease, suggesting that the optimal gas flow rate should be determined by research in practical applications. The changes of pH and σ in the reaction solution were tracked experimentally. It was demonstrated that ·OH and H_2_O_2_ produced in the reaction solution would play an important role in the degradation process. Molecular dynamics simulations demonstrated the degradation of the ERY by ROS, revealing the ring-opening reaction at two specific sites and the disruption of the branched chain, which greatly compromised the structural integrity of ERY, and dose simulations demonstrated that the reactivity of ·OH was greater than that of H_2_O_2_. In vitro antimicrobial experiments of *Staphylococcus aureus* proved that the antimicrobial effect of plasma-treated ERY solution was significantly reduced, and the treatment was safe and practical.

## Figures and Tables

**Table 1:** First-order kinetic equations for degradation.

Initial Concentration	k	R
250	−0.03794	0.98
350	−0.03491	0.99
450	−0.03091	0.98

**Table 2:** Reaction mechanism.

Equation of a Chemical Reaction	Serial Number
e + H_2_O → e + H^+^ + ·OH(aq)	(1)
e + H_2_O → H_2_O^+^ + 2e	(2)
e + H_2_O^+^ → 2e + H^+^ + ·OH(aq)	(3)
e + H_2_O → 2e + H^+^ + ·OH(aq)	(4)
Ar*(g) + H_2_O → Ar(g) + H^+^ + ·OH(aq)	(5)

**Table 3:** Structures mentioned in references [50,51].

Code	Chemical Structure
Compound D Product 4	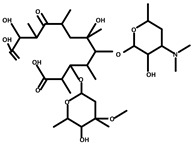
Product 8	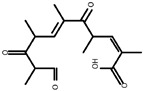

## Data Availability

Data are included in the article. Cartographic data can be viewed at the following link https://zenodo.org/records/14752222 (accessed on 1 January 2025).
